# Creatine ingestion augments dietary carbohydrate mediated muscle glycogen supercompensation during the initial 24 h of recovery following prolonged exhaustive exercise in humans

**DOI:** 10.1007/s00726-016-2252-x

**Published:** 2016-05-19

**Authors:** Paul A. Roberts, John Fox, Nicholas Peirce, Simon W. Jones, Anna Casey, Paul L. Greenhaff

**Affiliations:** 1 School of Biomedical Sciences, Queen’s Medical Centre, University of Nottingham, Nottingham, NG7 2UH UK; 2 Human Metabolic Physiology and Nutrition, QinetiQ Centre for Human Sciences, Farnborough, UK; 3 School of Life Sciences, The Medical School, Nottingham, NG7 2UH UK

**Keywords:** Glycogen storage, Glucose tolerance, Phosphocreatine, Insulin sensitivity

## Abstract

Muscle glycogen availability can limit endurance exercise performance. We previously demonstrated 5 days of creatine (Cr) and carbohydrate (CHO) ingestion augmented post-exercise muscle glycogen storage compared to CHO feeding alone in healthy volunteers. Here, we aimed to characterise the time-course of this Cr-induced response under more stringent and controlled experimental conditions and identify potential mechanisms underpinning this phenomenon. Fourteen healthy, male volunteers cycled to exhaustion at 70 % *V*O_2peak_. Muscle biopsies were obtained at rest immediately post-exercise and after 1, 3 and 6 days of recovery, during which Cr or placebo supplements (20 g day^−1^) were ingested along with a prescribed high CHO diet (37.5 kcal kg body mass^−1^ day^−1^, >80 % calories CHO). Oral-glucose tolerance tests (oral-GTT) were performed pre-exercise and after 1, 3 and 6 days of Cr and placebo supplementation. Exercise depleted muscle glycogen content to the same extent in both treatment groups. Creatine supplementation increased muscle total-Cr, free-Cr and phosphocreatine (PCr) content above placebo following 1, 3 and 6 days of supplementation (all *P* < 0.05). Creatine supplementation also increased muscle glycogen content noticeably above placebo after 1 day of supplementation (*P* < 0.05), which was sustained thereafter. This study confirmed dietary Cr augments post-exercise muscle glycogen super-compensation, and demonstrates this occurred during the initial 24 h of post-exercise recovery (when muscle total-Cr had increased by <10 %). This marked response ensued without apparent treatment differences in muscle insulin sensitivity (oral-GTT, muscle GLUT4 mRNA), osmotic stress (muscle *c*-*fos* and HSP72 mRNA) or muscle cell volume (muscle water content) responses, such that another mechanism must be causative.

## Introduction

Muscle glycogen availability has long been cited as a principal determinant of endurance exercise performance, and its depletion corresponds with the development of muscle fatigue (Bergström and Hultman 1967; Bergström et al. 1967). Optimising liver and muscle glycogen storage is a goal of many athletes wishing to maximise performance, particularly those involved in disciplines requiring prolonged sub-maximal exertion (65–75 % *V*O_2peak_) or repeated bursts of high intensity exercise. The accepted method of maximising muscle glycogen content is by ‘carbohydrate (CHO) loading’, which classically involves the depletion of muscle glycogen reserves through prolonged sub-maximal exercise (>90 min), followed by the consumption of a high CHO diet (>70 % calories CHO) for several days (Bergström and Hultman 1966, 1967; Sherman and Costill 1984). The use of such methods has been shown to replenish muscle glycogen content to a habitual resting level within 24 h, and to super-compensate reserves by over 100 % within 48–72 h. As a result of this, improvements in subsequent endurance exercise performance (time to fatigue*)* of about 50 % can be expected (Bergström and Hultman 1967). This super-compensatory response is confined solely to the previously exercised (glycogen-depleted) muscle, and has been attributed to heightened muscle insulin sensitivity and a localised increase in glycogen synthase activity (Bergström and Hultman 1967, 1966; Jentjens and Jeukendrup 2003).

We have previously demonstrated that dietary Cr supplementation can augment post-exercise muscle glycogen storage during a conventional ‘CHO-loading’ regimen in healthy, young, male volunteers, and that this response is restricted to the previously exercised limb (Robinson et al. 1999; Sewell et al. 2008). Furthermore, in keeping with the data of Harris et al. (1992), we confirmed that exercise can augment muscle Cr storage (Robinson et al. 1999). Of practical importance, this Cr-mediated augmentation of post-exercise muscle glycogen storage was of a magnitude sufficient to produce a significant improvement in endurance exercise performance (~150 mmol kg^−1^ dry muscle; Robinson et al. 1999). Although Cr ingestion is well established as a method to improve performance during, and recovery from (in the form of PCr re-synthesis), short-term maximal exercise (Greenhaff et al. 1993, 1994), the work of Robinson et al. (1999) highlighted for the first time the potential beneficial effect of Cr supplementation on the performance of, and maximising recovery from, prolonged sub-maximal exercise. The findings of Robinson et al. (1999) have since been supported by rodent (Op’t Eijnde et al. 2001a) and human (Op’t Eijnde et al. 2001b; Derave et al. 2003; Van Loon et al. 2004) based investigation. The time-course of this Cr-mediated effect on glycogen storage remains unknown, but based on the findings of Robinson et al. (1999) must occur somewhere between 6 and 120 h of post-exercise Cr ingestion. Similarly, insight is lacking into the mechanisms that underpin this phenomenon.

There are a number of potential mechanisms that might underpin the ability of dietary Cr supplementation to augment post-exercise muscle glycogen storage. First, a Cr-induced increase in post-exercise insulin release and/or increase in muscle insulin sensitivity. This hypothesis could be tested by performing an oral-glucose tolerance test (oral-GTT) before exercise and at several pre-determined time points during recovery from exercise, during which volunteers ingest Cr and CHO or CHO alone, and blood glucose and serum insulin are determined. Furthermore, depending upon the time-course of the glycogen super-compensatory response, a Cr-induced augmentation in muscle sarcolemmal glucose transporter (GLUT4) might occur in tandem with these responses (Op’t Eijnde et al. 2001b). Second, an increase in post-exercise glycogen storage could be directly linked to a Cr-induced increase in muscle water content. Indeed, osmotically induced swelling of rat muscle fibres in vitro has demonstrated that muscle glycogen synthesis can be increased by changes in cell volume (Low et al. 1996). Taken in tandem with the decreases in urinary volume and increases in fat-free body mass often reported following Cr ingestion (Hultman et al. 1996; Dentowski et al. 1997), if muscle Cr accumulation (which is sodium dependent) does result in cell swelling in vivo in humans (Francaux and Poortmans 1999), then this may indeed underpin an increase in post-exercise glycogen storage. This could be tested by determining Cr-mediated alterations in muscle water content in tandem with changes in the expression of transcription factors known to be sensitive to osmotic stress responses in humans, for example, *c*-*fos* and the inducible 70 kDa heat shock protein (HSP72; Locke 1997; Sadoshima and Izumo 1997; Beck et al. 2000). Finally, given exercise-induced muscle glycogen depletion is known to accompany increased muscle 5′AMP activated protein kinase (AMPK) activation, which in turn has been linked to the mechanistic regulation of post-exercise muscle glucose disposal and storage (Wojtaszewski et al. 2003; Steinberg et al. 2006), it is plausible that a Cr-mediated alteration in exercise-induced AMPK activation could modulate post-exercise muscle glycogen storage. Whilst AMPK is principally activated through changes in the AMP/ATP ratio (Hardie 2004; Hardie and Hawley 2001), a change in the PCr/Cr ratio, which is known to accompany Cr feeding in human muscle (Harris et al. 1992; Robinson et al. 1999), is also known to modulate AMPK activity (Ponticos et al. 1998). Furthermore, AMPK has been reported to be activated indirectly by changes in cell osmotic stress (Hayashi et al. 2000; Fryer et al. 2002).

The primary aim of the present study, therefore, was to extend the findings of Robinson et al. (1999) and to delineate the time-course of Cr-mediated post-exercise muscle glycogen super-compensation in humans, but under more stringent and controlled experimental conditions. We also hoped to provide some insight into potential mechanisms that could underpin this Cr-mediated phenomenon.

## Methods

### Subjects

Fourteen recreationally active (non-highly trained) and non-vegetarian healthy men (age 26 ± 2 years; height 180 ± 1 cm; body mass 78.6 ± 3.9 kg; body mass index 24.5 ± 1.0 kg m^−2^; *V*O_2peak_ 44.4 ± 1.5 ml kg^−1^ body mass min^−1^), with no history of prior Cr supplementation, volunteered to participate in the present study. The study was approved by the University of Nottingham Medical School Ethics Committee and conformed to the Declaration of Helsinki. Subjects were advised of the rationale, associated risks and procedures of the study and were aware that they were free to withdraw from the study at any time. Before commencing the investigation, all subjects gave informed written consent, and routine blood and physiological measurements were performed to assess health status prior to acceptance into the study.

### Maximal oxygen uptake determination

Upon satisfying the screening criteria, each subject visited the laboratory on two occasions during which they were familiarised with the experimental procedures to be used throughout the study and their peak oxygen uptake (*V*O_2peak_) was measured and confirmed. The determination of *V*O_2peak_ involved the use of an online gas analysis system (SensorMedics, Anaheim, CA, USA) and a continuous incremental exercise protocol on an electrically braked bicycle ergometer at a pedalling cadence of ~70 rpm (Excaliber Sport, Lode N.V. Instrumenten, Groningen, The Netherlands).

### Experimental protocol (Fig. 1)

Subjects reported to the laboratory on the morning of the study after an overnight fast, having abstained from alcohol and strenuous exercise for a minimum of 48 h. Upon arrival, subjects were weighed and then rested in a supine position with their non-dominant hand placed in a hand-warming unit (in which air temperature was maintained at 55 °C) to arterialise the venous drainage of the hand (Gallen and Macdonald 1990). After 20 min, a cannula was inserted into an antecubital vein on the dorsal surface of the subject’s hand, and the hand was returned to the hand-warming unit. The cannula was kept patent using an isotonic saline drip. Subjects then underwent a 2-h oral-glucose tolerance test (GTT), where 90 g of simple CHO was ingested in a 500 ml solution (<2 min), with arterialised-venous blood samples being taken immediately prior to CHO ingestion and 15, 30, 45, 60, 80, 100 and 120 min post-ingestion for the subsequent analysis of the whole blood glucose and lactate (YSI 2300 Statplus analyser, YSI, Yellow Springs, OH, USA), non-esterified free fatty acid (NEFA C kit, Wako Chemicals, Wako, Germany) and serum insulin (Diagnostic Products, Los Angeles, CA, USA) concentrations. Throughout the oral-GTT subjects continued to rest in a supine position and their hand remained in the hand-warming unit to ensure venous arterialisation was maintained.

**Fig. 1:**
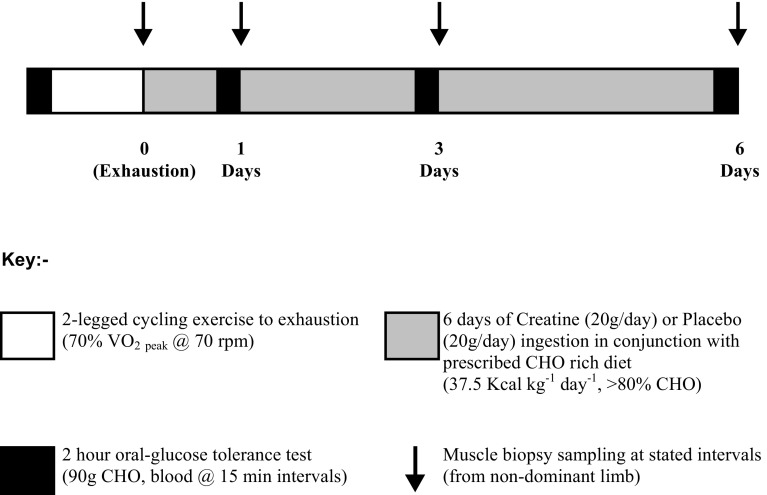
Diagrammatic overview of the experimental protocol

Upon completion of the oral-GTT, the cannula was removed and subjects immediately began exercising on an electrically braked bicycle ergometer (Excaliber Sport, Lode N.V. Instrumenten, Groningen, The Netherlands) at 70 % *V*O_2peak_ and a pedal cadence of 70 rpm. Expired gas composition and volume was measured throughout the first 15 min of exercise to confirm subjects were exercising at the correct workload. Perceived exertion was assessed every 15 min during exercise using the Borg scale (Borg 1982). To prevent excessive dehydration during exercise, subjects received 2 ml of water kg^−1^ body mass every 20 min throughout the exercise period. Subjects exercised continuously until they could no longer maintain a cycling cadence of 70 rpm and wished to stop. At this point, they were allowed to rest supine on a bed for 5 min, and then continued to exercise, interleaved with short rest periods, until the required cycling cadence could no longer be maintained for 2 min or the subject chose not to continue (point of exhaustion). This glycogen-depleting protocol has been used previously by this research laboratory to achieve almost complete muscle glycogen depletion (Casey et al. 1996).

### Muscle biopsy sampling and treatment groups (Fig. 1)

At the point of exhaustion, subjects stepped away from the exercise apparatus and rested supine on a bed, whilst a muscle biopsy was immediately obtained from the vastus lateralis of the non-dominant leg using the Bergström needle biopsy technique (Bergström 1975). Upon removal from the limb, the muscle biopsy sample was snap frozen in liquid nitrogen and stored under liquid nitrogen for analysis at a later date. Subjects were then randomly assigned to either a Cr monohydrate (20 g day^−1^) or placebo (glycine 20 g day^−1^) treatment group for the remainder of the day and subsequent 5 days. A further muscle biopsy, followed immediately by an oral-GTT, was performed after 1, 3 and 6 days of Cr and placebo ingestion. To avoid potential confounding metabolic effects arising from multiple biopsy sampling over the course of the study, biopsy sites were separated by at least 2.5 cm (Murton et al. 2014).

The Cr treatment group (*n* = 7) ingested 5 g of Cr (Cr monohydrate; AlzChem, Trostberg, Germany) on four equally spaced occasions each day over a 6 day period, whilst the placebo group (*n* = 7) received 5 g of glycine (Ajinomoto Inc., Tokyo, Japan) at the same time points. Each 5 g supplement (dissolved in 250 ml of a warm, sugar-free, diluted orange drink) was consumed, and immediately followed by the ingestion of 500 ml of a commercially available CHO-rich drink (Lucozade Original ≈18.5 % w/v glucose and simple sugars, Smith-Kline Beecham, Coleford, UK). This supplementation protocol ensures each Cr supplement is fully dissolved, and the peak in plasma Cr concentration coincides with peak elevations in blood glucose and serum insulin concentration (Green et al. 1996a, b). The CHO dose was chosen because it is known to stimulate muscle Cr and glycogen accumulation in young, healthy volunteers (Green et al. 1996a, b; Robinson et al. 2000). Supplement ingestion commenced immediately after the post-exercise muscle sample (1st biopsy). Before leaving the laboratory, subjects consumed a second treatment (supplement), as detailed previously, and a further two supplements were ingested at intervals over the remainder of that day. Typically, the supplements were ingested at ~12, 2, 6 and 10 pm daily. Subjects repeated the ingestion of the supplements four times each day for a further 5 days. In addition to the supplements, subjects consumed a commercially prepared CHO-rich diet (‘Be Good To Yourself™’ food range, Sainsburys, UK), which when added to the 2 l of Lucozade consumed each day, equated to greater than 80 % of the calorific intake being in the form of CHO throughout the 6 day treatment period (~8 g CHO kg^−1^ day^−1^). As subjects received 1350 kcal per day from the Lucozade drinks alone, to ensure that protein intake was not reduced from recommended levels, subjects received 20 % more calories per day than required based on their body mass (37.5 kcal kg^−1^ body mass day^−1^). This regimen of Cr ingestion has been widely used and has been proven to have no adverse side effects (Robinson et al. 2000), to increase plasma Cr concentration to a peak of ~700 µmol l^−1^ when co-ingested with CHO (Green et al. 1996a) and to markedly increase muscle total-Cr content.

Twenty-four h urine collections were made over the first day of supplementation (5 l containers, containing 5 ml of 0.6 mol l^−1^ thymol solution). Urine volume was recorded and following mixing; an aliquot was removed and stored at −80 °C for future HPLC analysis of urinary Cr and creatinine content (Dunnett et al. 1991).

### Muscle sample treatment and analysis

All biopsy samples were divided into two equal portions under liquid nitrogen. Subsequently, one portion was freeze-dried, dissected free from visible blood and connective tissue and powdered. Total muscle water content of samples was determined by weighing the samples before and after freeze-drying. Freeze-dried samples were then extracted in 0.5 M perchloric acid containing 1 mmol EDTA, with the resulting supernatant neutralised with 2.2 M KHCO_3_ and used for the spectrophotometric determination of glucose-6-phosphate (G-6-P), ATP, PCr and Cr (Harris et al. 1974). Freeze-dried muscle powder was also used for the determination of muscle glycogen (Harris et al. 1974).

Total-RNA was extracted from the remaining portion of frozen (wet) muscle using the method of Chomczynski and Sacchi (1987) and quantified using a kit (Molecular Probes RNA quantification reagent and kit, Cambridge BioScience, Cambridge, UK). Following this, 3 µl of total-RNA was diluted in 0.05 µg µl^−1^ RNA solution for the subsequent analysis of gene expression.

Following this, 0.5 µg of RNA was added to an Eppendorf tube containing random-hexamers (Promega, Southampton, UK) and RNase-free water, mixed and incubated within a thermal cycler (Mastercycler^®^ Gradient, Eppendorf, Hamburg, Germany) for 5 min at 70 °C and then placed on ice. Reverse transcriptase buffer, dATP, dCTP, dGTP, dTTP and dUTP mix, ribonuclease inhibitor, MMLV reverse transcriptase (Promega, Southampton, UK) and RNase-free water were then added to each sample. Samples were then mixed and incubated at 42 °C for 1 h. Following incubation, α-actin, GLUT4, *c*-*fos* and HSP72 mRNA was quantified by real-time PCR using an ABI PRISM 7700 Sequence Detector (Applied Biosystems, Warrington, UK). Probes and primers were designed using Primer Express™ software Version 2.0 (Applied Biosystems, Warrington, UK; Table 1), and all samples were run in triplicate. Results were expressed as ratios towards α-actin, which was considered an endogenous mRNA control allowing variation due to RNA extraction as well as RT (cycle times) efficiencies to be taken into account. Final normalising involved attributing a value of 100 % to the average of the triplicate of determinations for the pre-supplementation (post-exercise) time point.

**Table 1:** Sequences of forward primers, reverse primers, probes and the GenBank/EMBL accession numbers of genes of interest investigated using real-time polymerase chain reaction technology

Gene of interest	Accession number	Forward primer (5′–3′)	Reverse primer (5′–3**′**)	Probe (5′-FAM to 3′TAMRA)
α-Actin	J00068	GTGGCCCTGGACTTCGAG	TTGCCGATGGTGATGACCT	N/A (*SYBR* ^*®*^ *Green Master Mix*)
GLUT4	HSIRG7	CCCACTCTCCCCTCCCTCTTCA	TCCAATCCCCCTTCTCTAGCA	TCCTCCCCACCTTCCCCAGACTCA
*c*-*fos*	ACM16287	CTTCCTGTTCCCAGCATCATC	GGCTCCCAGTCTGCTGCATA	CCGCTCCGTGCCAGACATGGAC
HSP72	M11717	ACCAAGCAGACGCAGATCTTC	GCCCTCGTACACCTGGATCA	CCTACTCCGACAACCAACCCGGG

### Calculations and statistics

All data are reported as means ± SEM. Comparisons between treatments, for both absolute concentrations and changes from basal, were carried out using the two-way analysis of variance (ANOVA) with repeated measures. When a significant *F* value was obtained (*P* < 0.05), an *LSD* post hoc test was used to locate any differences (SPSS Base 8.0). Significance was accepted at the 5 % level, unless otherwise stated in the text.

With the exception of lactate, the content of all muscle metabolites was adjusted to the mean ATP concentration within each individual (based upon four biopsy samples). By this means, it was possible to compensate for any admixture of connective tissue and other non-muscular elements within each muscle biopsy sample (Harris et al. 1992).

Glucose, lactate, insulin and non-esterified fatty acid data (NEFA), shown as area under the curve, were calculated from the individual glucose, lactate, serum insulin and NEFA blood concentrations of each subject at ~15 min intervals (0, 15, 30, 45, 60, 80, 100 and 120 min) during the 120 min of the oral-GTT on the treatment days indicated (Kaleidagraph, Synergy Software, Reading, USA).

## Results

Subjects reported compliance with all exercise, supplementation and dietary aspects of the study and did not report any ill effects. Maximal oxygen uptake, daily energy intake and dietary composition were identical between treatment groups (Table 2). Urinary Cr excretion was negligible during the first 24 h of placebo supplementation. However, Cr excretion increased dramatically during day 1 of Cr supplementation and was significantly greater than that observed in the placebo group (Cr 0–24 h = 7.6 ± 1.5 g vs. placebo 0–24 h = 0.0 ± 0.4 g; *P* < 0.01), pointing to ~60 % of the 20 g Cr ingested being retained by the body during the first 24 h of supplementation. No differences in urinary creatinine excretion existed between groups during the first day of Cr or placebo supplementation (Cr 0–24 h = 1.2 ± 0.3 g vs. placebo 0–24 h = 0.9 ± 0.3).

**Table 2:** Peak oxygen consumption (*V*O_2peak_), daily energy intake and dietary composition of the placebo (glycine) and creatine treatment groups

	Placebo (*n* = 7)	Creatine (*n* = 7)
*V*O_2peak_ (ml kg^−1^ min^−1^)	45.9 ± 2.3	42.9 ± 2.0
Energy intake (kcal kg^−1^ day^−1^)	37.6 ± 0.0	37.5 ± 0.0
Carbohydrate (g kg^−1^ day^−1^)	8.1 ± 0.0	8.2 ± 0.1
Carbohydrate (% of calories)	80.8 ± 0.3	81.9 ± 0.5
Protein (% of calories)	13.5 ± 0.4	13.0 ± 0.3
Fat (% of calories)	7.5 ± 0.1	7.0 ± 0.4

Values expressed as mean ± SEM

No difference in muscle water content from pre-supplementation (post-exhaustive exercise) existed within or between treatment groups throughout 6 days of placebo or Cr supplementation (Table 3).

**Table 3:** Muscle water content and metabolite concentrations in exercised human vastus lateralis muscle before (exhaustion) and after 1, 3 and 6 days of creatine + carbohydrate (*n* = 7) or placebo + carbohydrate (*n* = 7) supplementation

Metabolite	Treatment	Exhaustion	1 Day	3 Days	6 Days
[H_2_O]	Placebo	77.4 ± 0.9	79.4 ± 2.4	78.0 ± 1.8	81.4 ± 2.9
	Creatine	80.5 ± 2.0	81.8 ± 2.0	78.1 ± 1.2	77.4 ± 1.0
[ATP]	Placebo	24.9 ± 0.8	24.6 ± 0.9	23.6 ± 0.9	23.3 ± 0.9
	Creatine	24.2 ± 1.0	23.8 ± 0.7	24.8 ± 0.9	24.8 ± 0.7
[G-6-P]	Placebo	4.4 ± 0.6	4.6 ± 0.6	5.8 ± 1.9	5.2 ± 3.1
	Creatine	4.8 ± 0.6	3.6 ± 0.6	4.1 ± 0.5	5.2 ± 0.5
[PCr]	Placebo	86.9 ± 2.4	87.0 ± 2.3	87.8 ± 2.1	85.1 ± 3.0
	Creatine	84.5 ± 4.5	91.8 ± 2.5	93.1 ± 2.5^†^	98.7 ± 2.5^††^**
[Creatine]	Placebo	41.1 ± 2.1	41.0 ± 2.1	43.1 ± 1.7	43.9 ± 2.3
	Creatine	42.6 ± 1.8	46.3 ± 2.0	51.7 ± 0.8^††^**	59.2 ± 2.4^††^**

All values are expressed as means ± SEM. Muscle water content expressed as a percentage of the total muscle sample weight. Muscle ATP, glucose-6-phosphate (G-6-P), phosphocreatine (PCr) and creatine are expressed as mmol kg^−1^ dry muscle (with the exception of lactate, the content of all muscle metabolites was adjusted to the mean ATP concentration within each individual (based upon four biopsy samples)

### Muscle metabolites

No change in muscle ATP or G-6-P content was observed from the pre-supplementation (post-exercise) time point during 6 days of Cr and placebo supplementation (Table 3). Similarly, no differences in muscle ATP and G-6-P content existed between treatment groups over the time-course of the study (Table 3).

No significant change in muscle PCr, free-Cr or total-Cr content over time from the pre-supplementation time point was observed with placebo supplementation (Table 3). There was a 17 % significant increase in muscle PCr content from the pre-supplementation value following 3 days of Cr supplementation (*P* < 0.05), which increased further following 6 days of Cr ingestion (*P* < 0.01, Table 3). Creatine ingestion increased muscle PCr content above placebo following 6 days of supplementation (*P* < 0.01, Table 3). Similarly, Cr ingestion increased muscle free-Cr content following 3 days of supplementation (*P* < 0.01, Table 3), which was 39 % (*P* < 0.01) greater than the pre-supplementation value after 6 days of Cr ingestion (Table 3). Creatine ingestion increased muscle free-Cr content above placebo after 3 days of supplementation (*P* < 0.01), which continued to increase to 35 % greater than placebo following 6 days of Cr ingestion (*P* < 0.01, Table 3).

**Fig. 2:**
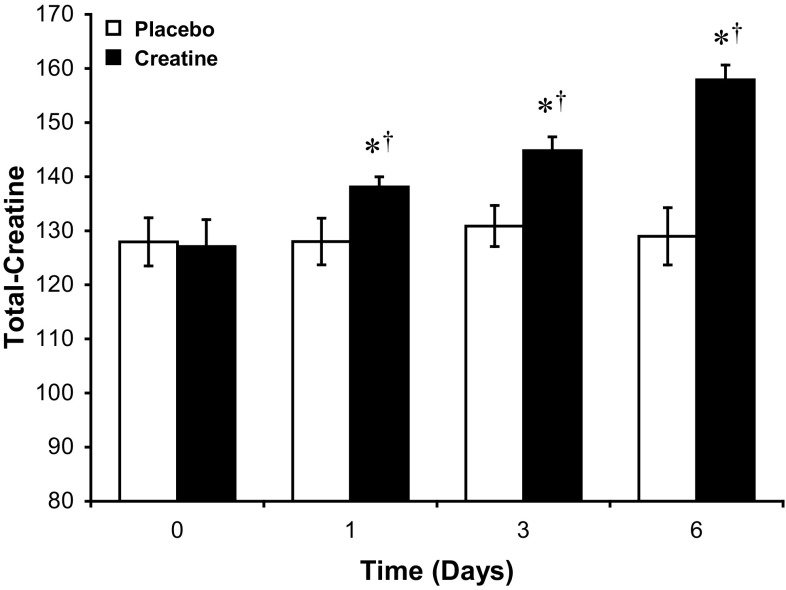
Skeletal muscle total-creatine (TCr) content during 6 days of creatine + carbohydrate (Creatine, *n* = 7) or glycine + carbohydrate (Placebo, *n* = 7) supplementation following glycogen-depleting exercise in man. Results are expressed as means ± SEM with units of mmol kg^−1^ dry muscle. Different from the pre-supplementation time point (post-exercise, time 0) within the same treatment group (^†^ *P* < 0.05, ^††^ *P* < 0.01); different from placebo at the corresponding time point (*P < 0.05, **P < 0.01)

In accordance with these changes, muscle total-Cr content was greater than the pre-supplementation value following 1 (9 %, *P* < 0.05), 3 (14 %, *P* < 0.01) and 6 (24 %, *P* < 0.01) days of ingestion (Fig. 2). Creatine ingestion increased muscle total-Cr content above placebo after 1 (8 %, *P* < 0.05), 3 (11 %, *P* < 0.01) and 6 (22 %, *P* < 0.01) days of Cr ingestion (Fig. 2).

No alteration in the PCr:Cr ratio occurred throughout the study in the placebo group. However, the PCr:Cr ratio was lower than the pre-supplementation value (2.00 ± 0.13) after 3 days Cr supplementation (1.81 ± 0.06, *P* < 0.05). This value was also lower than that recorded in the placebo group after 3 days supplementation (2.05 ± 0.03, *P* < 0.05).

**Fig. 3:**
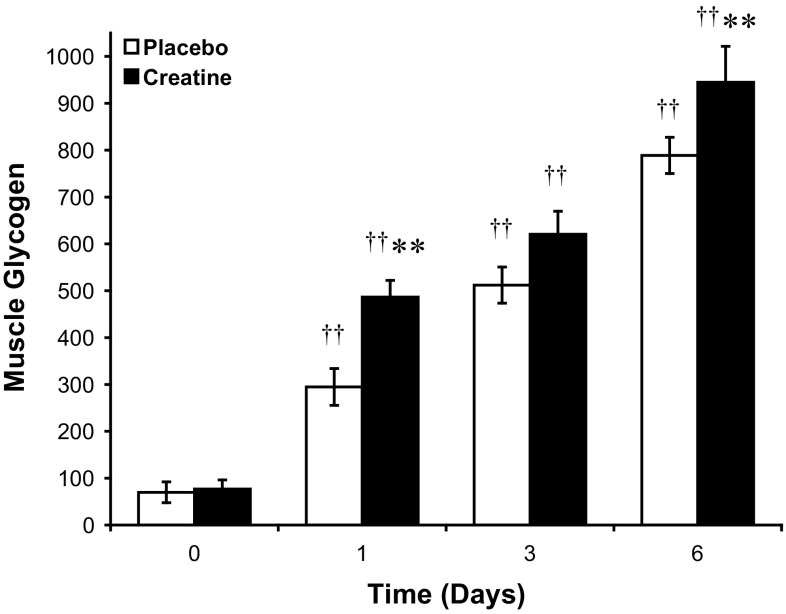
Skeletal muscle glycogen content during 6 days of creatine + carbohydrate (Creatine, *n* = 7) or glycine + carbohydrate (Placebo, *n* = 7) supplementation following glycogen-depleting exercise in man. Results are expressed as means ± SEM with units of mmol kg^−1^ dry muscle. Different from the pre-supplementation (post-exercise, time 0) time point within the same treatment group (^†^ *P* < 0.05, ^††^ *P* < 0.01); different from placebo at the corresponding time point (*P < 0.05, **P < 0.01)

The exercise intervention markedly reduced skeletal muscle glycogen content from the habitual resting value of ~400–450 mmol kg^−1^ dry muscle, and there was no difference in the post-exercise value between treatment groups immediately prior to Cr and placebo ingestion (Fig. 3). Muscle glycogen content increased dramatically in both treatment groups during 6 days of supplementation, and as expected muscle glycogen content was greater than the post-exercise value in both treatment group following 1, 3 and 6 days (Fig. 3). However, Cr supplementation increased muscle glycogen content significantly above placebo after 1 (*P* < 0.01) and 6 (*P* < 0.01) days (Fig. 3), with the augmentation of glycogen storage being almost exclusively confined to the initial 24 h of Cr supplementation, and the difference between treatments being maintained thereafter (*P* < 0.01, Fig. 3). Indeed, the magnitude of glycogen re-synthesis during the first 24 h of supplementation was ~82 % greater in the Cr group compared to placebo (Cr 410 ± 50 vs. placebo 225 ± 50 mmol kg^−1^ dry muscle, *P* < 0.01), with no difference in the rate of glycogen synthesis existing between groups between 1 and 6 days of supplementation (Fig. 3).

### Blood metabolites and oral-glucose tolerance tests

**Fig. 4:**
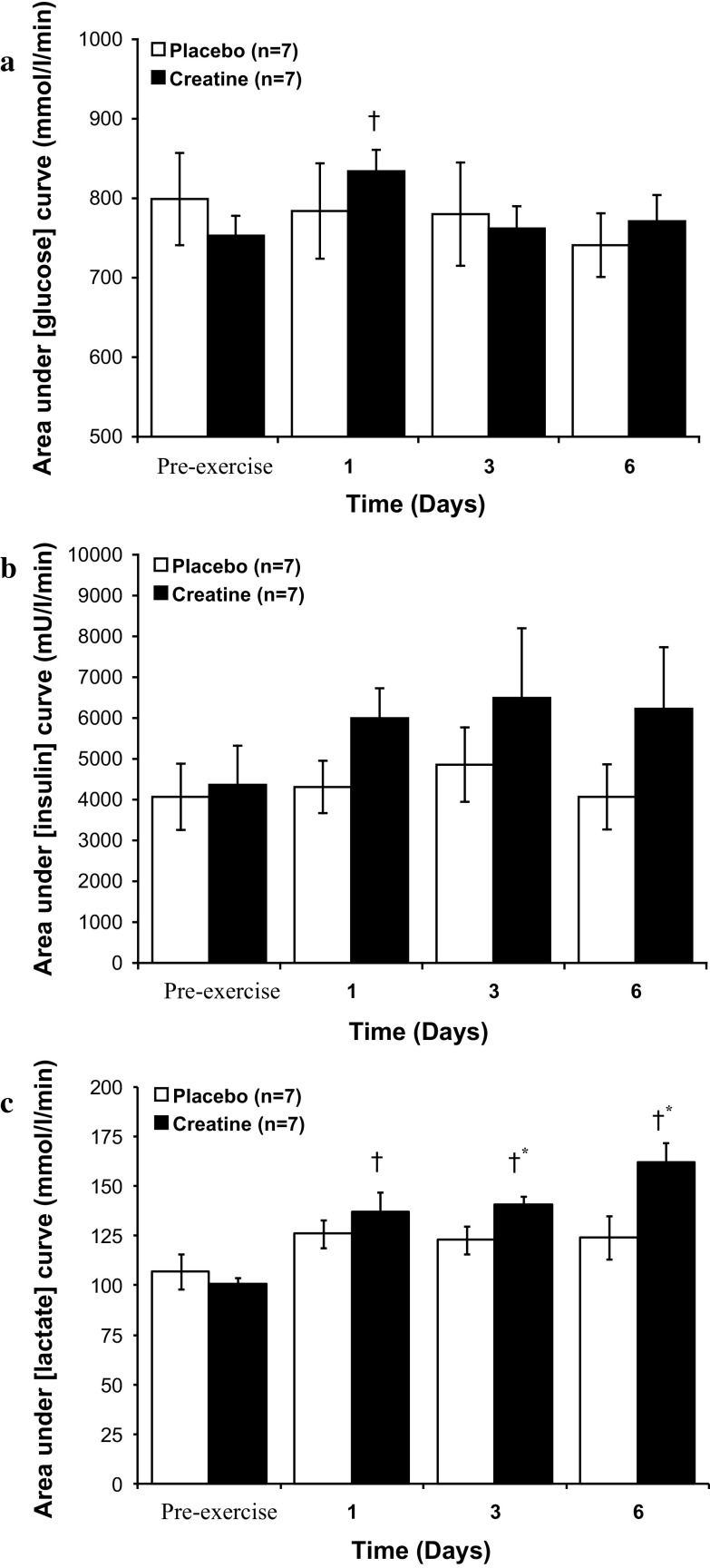
Area under the curve during an oral glucose tolerance test (GTT) for blood glucose (**a**), serum insulin (**b**) and blood lactate (**c**) before (pre-exercise) and after 1, 3 and 6 days of Cr + carbohydrate or glycine + carbohydrate supplementation following glycogen-depleting exercise in man. Results are expressed as means ± SEM. Area under plasma glucose and lactate curve expressed as (mmol l^−1^ min^−1^), area under serum insulin curve expressed as (mU l^−1^ min^−1^). *†* different from the pre-exercise time point within the same treatment group (*P* < 0.05); * different from placebo group at the corresponding time point (*P* < 0.05)

The area under the blood glucose-time curve during the oral-GTT (defined in Methods) was unchanged from the pre-supplementation value throughout 6 days of placebo ingestion (Fig. 4a). Creatine supplementation transiently elevated the area under the glucose curve from basal after 1 day of ingestion (*P* < 0.05, Fig. 4a), but no differences in the area under the curve existed between treatment groups at any time point throughout the study (Fig. 4a).

Neither placebo nor Cr ingestion had an effect on the area under the serum insulin-time curve during the oral-GTT throughout the study (Fig. 4b). Similarly, no difference between treatment groups was observed at any time point during 6 days of ingestion (Fig. 4b). There was a weak trend for Cr supplementation to increase the serum insulin concentration above placebo after 1 day of supplementation (Placebo day 1 = 4310 ± 642 vs. Cr day 1 = 5995 ± 733 mU/l/min, *P* = 0.11, Fig. 4b).

The area under the blood lactate-time curve during the oral-GTT was unchanged from pre-supplementation throughout 6 days of placebo ingestion (Fig. 4c). Creatine supplementation increased the area under the blood lactate-time curve from pre-supplementation after 1 (*P* < 0.05), 3 (*P* < 0.05) and 6 (*P* < 0.05) days of ingestion (Fig. 4c). The area under the blood lactate-time curve was also elevated above placebo after 3 (*P* < 0.05) and 6 (*P* < 0.05) days of Cr ingestion (Fig. 4c).

Placebo and Cr supplementation both caused a significant reduction in area under blood non-esterified free fatty acid-time curve during the oral-GTT (data not shown). However, no difference existed between treatment groups at any point over the course of the study.

### mRNA expression

No differences in GLUT4 mRNA expression existed between placebo and Cr treatment groups throughout 6 days of supplementation (Table 4). However, GLUT4 mRNA expression increased ~twofold from the post-exercise time point following 1 and 6 days of placebo supplementation (*P* < 0.05; Table 4). No alteration in GLUT4 expression was observed from the post-exercise time point throughout 6 days of Cr supplementation (Table 4).

**Table 4:** Percentage change in skeletal muscle GLUT4, *c*-*fos* and HSP72 expression from immediately post-glycogen-depleting exercise (set at 100 %) after 1, 3 and 6 days of creatine + carbohydrate (*n* = 7) or placebo + carbohydrate (*n* = 7) supplementation

mRNA	Treatment	1 Day	3 Days	6 Days
GLUT4	Placebo	201.8 ± 57.2^†^	164.2 ± 43.5	210.4 ± 57.0^†^
	Creatine	131.6 ± 15.7	134.6 ± 32.1	123.8 ± 14.2
*c*-*fos*	Placebo	18.2 ± 8.7^††^	37.5 ± 18.0^††^	20.5 ± 10.8^††^
	Creatine	23.4 ± 8.7^††^	49.1 ± 18.3^††^	26.9 ± 16.1^††^
HSP72	Placebo	74.1 ± 15.5	49.5 ± 9.6^†^	75.4 ± 24.6
	Creatine	71.4 ± 18.9	49.7 ± 14.3^†^	48.6 ± 12.9^†^

Values are means ± SEM and are relative to the endogenous control (α-actin) mRNA level and expressed as a percentage of the corresponding pre-supplementation value. Different from pre-supplement time point (^†^ *P* < 0.05, ^††^ *P* < 0.01)

*c*-*fos* mRNA expression was markedly reduced from the post-exercise (pre-supplementation) time point throughout 6 days of supplementation in both treatment groups (*P* < 0.01; Table 4) However, no differences in expression were evident between treatment groups throughout the study (Table 4).

No differences in HSP72 mRNA expression existed between treatment groups throughout 6 days of supplementation (Table 4). However, HSP72 expression was reduced from the post-exercise time point following 3 days of supplementation in both treatment groups (*P* < 0.05; Table 4). The reduced expression of HSP72 was transient in the placebo group, but was sustained after 6 days of Cr supplementation (*P* < 0.05; Table 4).

## Discussion

The primary aim of the present study was to delineate the time-course of dietary Cr-mediated post-exercise muscle glycogen super-compensation in humans under conditions, where energy intake and food consumption were stringently controlled. The principal finding was that dietary Cr supplementation markedly augmented post-exercise muscle glycogen storage above placebo during a conventional ‘carbohydrate-loading’ regime, and that this augmentation of glycogen storage occurred almost exclusively within the first 24 h of supplementation (the magnitude of glycogen re-synthesis during the first 24 h of supplementation was ~82 % greater in the Cr group compared to placebo). The results also point to this Cr-induced glycogen super-compensation being independent of the time-course of muscle Cr accumulation and changes in serum insulin availability, insulin sensitivity and/or cellular osmotic stress responses, such that another mechanism(s) must be causative.

### Muscle creatine and glycogen storage

Several studies have evaluated muscle glycogen storage in humans in response to acute (20 g day^−1^ for 5 days; Robinson et al. 1999; Newman et al. 2003; van Loon et al. 2004) and more prolonged Cr ingestion (2.5–20 g day^−1^ for 8–12 weeks; Op’t Eijnde et al. 2001b; Derave et al. 2003). The findings of these studies largely support a role for dietary Cr in enhancing muscle glycogen storage (+12–23 %) when ingested following exhaustive exercise (Robinson et al. 1999), or during 6–8 weeks of resistance training (+30–35 %; Op’t Eijnde et al. 2001b; Derave et al. 2003). The evidence that dietary Cr ingestion can augment muscle glycogen storage in the non-exercised state is less compelling, with a positive effect (+18 %) being reported by van Loon et al. (2004) following 5 days of Cr supplementation, but no effect at all being reported by others (Newman et al. 2003; Sewell et al. 2008), even within the same volunteer when one limb has been exercised and the contra-lateral limb has remained inactive (Robinson et al. 1999). As far as we are aware, the present study is the first to establish the post-exercise glycogen super-compensatory properties of dietary Cr supplementation under rigorously controlled dietary conditions in humans (Table 2) and to document the time-course of this effect. It is clear from the data (Fig. 3) that the Cr-induced augmentation of muscle glycogen storage occurs not as a gradual bifurcation from the typical glycogen-loading response observed in the placebo group, but rather as a transient marked increase (approximately twofold) in glycogen re-synthesis during the initial 24 h of Cr ingestion during recovery from exercise (Fig. 3).

It has been suggested that the expansion of the muscle total-Cr pool is a prerequisite for Cr-induced augmentation of muscle glycogen storage (Van Loon et al. 2004; Volek and Rawson 2004). Indeed, work by Van Loon et al. (2004) showed a significant correlation between the magnitude of increase in muscle total-Cr (+31 %) and glycogen storage (+18 %) following 5 days of Cr supplementation. This observation was strengthened by the findings from another study of this research group, where the absence of a treatment effect upon glycogen storage was attributed to inadequate muscle Cr accumulation (+12 %; Newman et al. 2003). However, in neither of these studies were muscle biopsies obtained over the time-course of Cr supplementation. Indeed, as the Cr-mediated augmentation of glycogen storage in the present study occurred almost exclusively within the first 24 h of Cr ingestion, when muscle total-Cr stores had increased by only 8 % (Fig. 2), the findings clearly do not support the contention that the extent of muscle total-Cr accumulation is an important determinant of the muscle glycogen storage response. Furthermore, following 24 h, no greater muscle glycogen storage above that recorded in the placebo group was observed from days 1 to 6 of Cr ingestion, despite muscle total-Cr increasing by a further 15 % in the Cr supplementation group (Figs. 2, 3).

It could be speculated that a transient change in the PCr/Cr ratio in response to 24 h of Cr feeding may have blunted the exercise-induced activation of AMPK (Winder and Hardie 1999; Rasmussen and Winder 1997; Fujii et al. 2000), which in turn, and independent of any alteration in muscle insulin sensitivity, could have augmented muscle glycogen synthesis during the initial 24 h of post-exercise recovery by dampening the inhibitory effect of AMPK on glycogen synthase activity (Aschenbach et al. 2002; Wojtaszewski et al. 2002; Jorgensen et al. 2004). Indeed, an alteration in the composition of the muscle Cr pool, as occurs, for example, during exercise and Cr feeding, has been reported to directly impact upon the activation status of AMPK (Ponticos et al. 1998). The in vitro findings of Ponticos et al. (1998) showed that physiological increases in muscle PCr content directly inhibited AMPK activation in a dose-dependent manner. Furthermore, this inhibition was attenuated (indirectly) by an increase in muscle free-Cr content; pointing to the PCr/Cr ratio, along with the ATP/ADP ratio, being a regulator of skeletal muscle AMPK activation status (for review, see Hardie and Hawley 2001). A reduction in the muscle PCr/Cr ratio has been reported following acute-Cr ingestion (Harris et al. 1992; Robinson et al. 1999), which has prompted researchers to examine the effect of Cr availability on AMPK activation in vitro (Ceddoa and Sweeny 2004) and in vivo (Ju et al. 2005; Op’t Eijnde et al. 2005). The findings from these studies are equivocal, with AMPK being activated by unphysiological concentrations of PCr and Cr (Ceddoa and Sweeny 2004), and independent of any alteration in the muscle PCr/Cr ratio (Ju et al. 2005), in some, but not all, studies (Op’t Eijnde et al. 2005). In the present in vivo study, Cr ingestion reduced the muscle PCr/Cr ratio from basal following 3 days of supplementation, but this response occurred after the Cr-mediated increase in muscle glycogen storage (0–24 h, Fig. 3), making it unlikely that this underpinned the augmentation of glycogen storage observed between 0 and 24 h. Furthermore, contrary to the report of Ponticos et al. (1998), more recent in vitro research has surmised that although the concept of a PCr/Cr ratio mediated regulation of AMP kinase may be intuitive and attractive, evidence to substantiate this concept is not prevalent (Taylor et al. 2006; Suter et al. 2006).

### Muscle creatine and insulin sensitivity

It is logical to assume that the rapid augmentation of post-exercise glycogen storage that accompanied Cr ingestion (Fig. 3) would have been preceded by an increase in muscle glucose uptake, which we hoped to demonstrate by performing oral-GTTs and determining muscle GLUT4 mRNA expression (Op’t Eijnde et al. 2001b) over the course of 6 days of exercise recovery. However, given that the Cr-induced augmentation of muscle glycogen storage occurred almost exclusively within the first 24 h of treatment and, therefore, preceded the first post-exercise oral-GTT (1 day), any effect of Cr ingestion on glucose clearance must have occurred within the initial 24 h of recovery. Indeed, based upon our previous observation (Robinson et al. 1999), which found no effect of Cr ingestion on muscle glycogen storage during the initial 6 h of recovery from exercise, and the present study that showed a dramatic effect after 24 h of recovery (Fig. 3), this Cr augmentation of glycogen storage must have occurred between 6 to 24 h of post-exercise recovery. The increase in area under the blood glucose curve during the oral-GTT after 1 day of Cr ingestion (Fig. 4a), and along with the trend for the serum insulin response to do the same (Fig. 4b), most probably reflects a blunting in muscle glucose uptake caused by the marked increase in muscle glycogen storage over the preceding 24 h. This view is supported by the increase in blood lactate concentration that accompanied Cr ingestion (Fig. 4c).

It has been suggested that Cr supplementation can up-regulate muscle GLUT4 protein expression, thereby increasing muscle glucose uptake and underpinning the glycogen super-compensatory properties of Cr supplementation (Op’t Eijnde et al. 2001b). However, this view is not supported by the findings others (Op’t Eijnde et al. 2001a; van Loon et al. 2004), where, for example, no increase in muscle GLUT4 mRNA or protein expression was observed in response to acute or prolonged Cr ingestion in healthy human volunteers (van Loon et al. 2004). The finding of the present study that Cr ingestion had no influence on muscle GLUT4 mRNA expression (Table 4) is in concordance with this stance. However, it is acknowledged that the quantification of muscle GLUT4 protein and/or components of the signalling cascade regulating GLUT4 translocation (e.g., AS160 activation) would have provided more robust insight.

### Muscle cell volume and osmotic stress responses

Another mechanism that has been suggested to underpin the stimulatory effect of Cr ingestion on acute glycogen super-compensation is an increase in intra-myocellular water content, secondary to an increase in muscle Cr accumulation (Robinson et al. 1999; Op’t Eijnde et al. 2001b; van Loon et al. 2004). Indeed, muscle Cr transport is dependent upon extracellular Na^+^, and is, therefore, osmotically active (Daly and Seifter 1980), with an increase in fat-free body mass and a decrease in urinary volume being reported in response to acute-Cr feeding; indicative of an increase whole-body water retention (Hultman et al. 1996). It has been demonstrated in vitro that changes in cell volume in rodent muscle can modulate changes in muscle glycogen content, independent of any alteration in cell glucose uptake (Low et al. 1996). However, although muscle total-Cr (Fig. 2) and glycogen (Fig. 3) both increased during the initial 24 h of dietary Cr ingestion in the present study, the time-course of change for each metabolite over the 6 days of supplementation was very different, with the glycogen super-compensatory response being almost exclusively restricted to the initial 24 h of supplementation, whilst muscle total-Cr content increased in a more gradual manner over the study. It is unlikely, therefore, that a Cr-mediated increase in cellular hydration status stimulated this marked initial increase in muscle glycogen storage, such that another mechanism seems likely. This conclusion is supported by the lack of any treatment effect on muscle water content during 6 days of supplementation (Table 3) and by the absence of any differences in mRNA expression of the osmotically induced transcription factors *c*-*fos* and HSP72 between Cr and placebo groups (Table 4). Changes in the expression of HSP72 (Febraio et al. 2002) and *c*-*fos* (Puntschart et al. 1998) mRNA have been shown to correlate well with changes in their respective protein products, indicating they are at least partly under transcriptional regulation. Furthermore, it has been suggested that muscle HSP72 expression is sensitive to alterations in myofibril glycogen content in man, with HSP72 mRNA increasing in response to glycogen-depleting exercise before gradually being reduced as glycogen stores are replenished (Febbraio and Koukoulas 2000; Febraio et al. 2002). Although the present study lacked a pre-exercise muscle biopsy, in support of the findings of Febraio et al. (2002), post-exercise HSP72 expression was halved following 3 days of recovery in the Cr and placebo groups, as muscle glycogen was super-compensated (Table 4). However, it is clear that HSP72 mRNA expression was insensitive to the marked differences in muscle glycogen content that existed between treatment groups following 1 day of supplementation (Fig. 3; Table 4).

In conclusion, we have confirmed the ability of dietary Cr supplementation to augment post-exercise muscle glycogen storage during a conventional and rigorously controlled ‘carbohydrate-loading’ regimen in humans. Furthermore, this Cr-induced augmentation of muscle glycogen storage occurred almost exclusively within the first 24 h of exercise recovery and Cr ingestion, and appeared to be unrelated to changes in the magnitude of increase in muscle total-Cr, PCr or Cr content, muscle insulin sensitivity, or osmotic stress and muscle cell volume responses, such that another mechanism must be causative.
